# Awake Craniotomy in Neurosurgery: A Bibliometric Analysis of the Top 100 Most-Cited Articles and Review of Technological Advancements

**DOI:** 10.7759/cureus.76290

**Published:** 2024-12-23

**Authors:** Billy McBenedict, Wilhelmina N Hauwanga, Yan Bin Fong, Anna Pogodina, Ebigbo E Obinna, Swetapadma Pradhan, Syeda Sukaina Kazmi, José Geraldo M Netto, Bruno Lima Pessôa

**Affiliations:** 1 Department of Neurosurgery, Universidade Federal Fluminense, Niterói, BRA; 2 Department of General Medicine, Universidade Federal do Rio de Janeiro, Rio de Janeiro, BRA; 3 Department of Surgery, Universiti Putra Malaysia, Seri Kembangan, MYS; 4 Faculty of Medicine, University of Buckingham, Buckingham, GBR; 5 Department of Public Health, Louisiana State University, Shreveport, USA; 6 Medical School, European University Faculty of Medicine, Tbilisi, GEO; 7 Medical School, Ziauddin Medical College, Ziauddin University, Karachi, PAK

**Keywords:** awake craniotomy, bibliometric analysis, eloquent brain regions, functional preservation, gender disparities in neurosurgery, glioma resection, intraoperative mapping, neuro-oncology, neurosurgical innovation, technological advancements

## Abstract

Awake craniotomy (AC) is a critical neurosurgical technique for maximizing tumor resection in eloquent brain regions while preserving essential neurological functions like speech and motor control. Despite its widespread adoption, no prior bibliometric analysis has evaluated the most influential research in this field. This study analyzed the top 100 most-cited articles on AC to identify key trends, influential works, and authorship demographics. A systematic search of the Web of Science Core Collection on September 17, 2024, yielded 718 publications, with the top 100 ranked by citation count. Analysis revealed a surge in AC research after 2013, peaking in 2021, with the *Journal of Neurosurgery* contributing significantly (49 articles; 2,611 citations). Themes included functional mapping, anesthetic techniques, and patient outcomes, with technological advancements such as intraoperative MRI and virtual reality enhancing surgical precision. Authorship analysis highlighted a gender disparity, with male authors occupying 77% of first authorship and 88% of senior roles. These findings underscore AC's evolution, foundational studies, and ongoing advancements while emphasizing the need for greater diversity and inclusion in the field.

## Introduction and background

Awake craniotomy (AC) with brain mapping is the gold standard for tumor or lesion resection in or near eloquent brain regions [1]. This technique minimizes neurological deficits by preserving essential functions like speech and motor control. The mapping process involves direct electrical stimulation of the cortex while the patient is awake and performing relevant tasks, helping identify if a particular stimulus disrupts the function being tested [1]. Advances in technology, such as the development of computerized platforms for behavioral testing, have further refined intraoperative mapping, particularly for language functions. These systems are designed to meet the specific environmental demands of the operating room and preoperative functional MRI (fMRI), ensuring better alignment between brain mapping techniques [2,3]. Over the past several decades, AC has become increasingly prevalent in neurosurgery, particularly for the removal of intrinsic brain tumors located in eloquent areas. This technique is primarily employed to preserve critical functions, such as motor and language abilities, by allowing direct interaction with the patient during surgery [4]. While awake resection has been extensively studied in the context of low-grade gliomas, the functional outcomes, particularly motor function, have been well-documented, correlating with the success of direct electrical stimulation (DES) in mapping essential regions [4]. Despite the significant progress in motor preservation, mapping other functions, such as language, poses unique challenges due to the complexity of localization.

Patient selection for AC is a critical process, requiring comprehensive psychological, cognitive, and functional assessments [5]. Psychological factors, such as anxiety and fear of pain, can significantly affect intraoperative compliance, potentially leading to failure [5]. In addition, medical considerations, including tumor location, anesthesia-related concerns, pregnancy status, and the patient's ability to cooperate, play essential roles in determining suitability for AC [1,6,7]. AC often reduces the need for postoperative intensive care monitoring, resulting in shorter or eliminated ICU stays. Compared to craniotomy under general anesthesia, patients undergoing AC experience fewer neurological deficits (7% vs. 23%) and shorter hospital stays (1.7 days vs. 9 days)[1]. These patients also report less postoperative pain, nausea, and vomiting [1]. 

While AC is generally safe for well-selected patients, contraindications, such as uncontrolled seizures, severe neurological deficits, or large tumors can necessitate alternative surgical approaches [1,3]. In addition, some studies have reported patients experiencing anxiety and depressive symptoms postoperative, often associated with pre-existing psychological conditions [8-10]. However, a systematic review reported that AC is generally well-tolerated and does not significantly increase stress, anxiety, or depression compared to general anesthesia, especially when performed by experienced teams [11]. Younger age and female sex may increase susceptibility to anxiety during the procedure, highlighting the need for personalized psychological preparation and support. Comprehensive psychiatric assessments across all phases of AC can enhance patient outcomes, emphasizing the importance of tailored care. Preoperative anxiety can influence postoperative pain perception and recovery outcomes. A study assessing 20 patients’ psychological states before, during, and after surgery found that while AC did not worsen anxiety or depression, preoperative anxiety correlated with higher postoperative pain and discomfort, particularly on the third day after surgery [10]. In studies assessing AC for glioma treatment in eloquent areas, patients generally tolerated mild postoperative deficits, such as facial motor dysfunction and verbal speed reduction, perceiving these as acceptable trade-offs for preserving other cognitive functions [12]. A case series in a multilingual Asian population further underscored that preoperative cognitive and emotional states directly influenced health-related quality of life (HRQoL) outcomes post surgery. Patients with better preoperative cognitive scores reported higher HRQoL, while moderate preoperative depression and stress were associated with poorer outcomes, suggesting the need for targeted perioperative psychological care [13].

Despite the increase in research and technological advances supporting AC, a comprehensive understanding of the most impactful contributions to this field remains limited. Therefore, the aim of this study was to conduct a bibliometric analysis of the top 100 most influential articles on AC and their author distribution based on sex, identifying key research trends, highly cited works, influential authors, and institutions. In addition, a review of the technological advancements supporting AC was performed. 

## Review

Material and methods

Data Sources and Search Strategies

The scientific papers were retrieved from the Web of Science Core Collection (WoSCC) on September 17, 2024. To accomplish a thorough analysis of the publications and prevent any fluctuations in citation counts, there were no restrictions on the publication year and the search and download were conducted on the same day. This study included articles, review articles, proceeding papers, and early access. The following keywords were applied on the search: (("Awake craniotomy") (Title) OR ("Awake craniotomy") (Abstract) Not ("Bibliometric Analysis") (Title)). A total of 718 publications were downloaded in "plain text" format, with the record content set to "full record and cited references." After screening, no duplicates were identified. As this study used secondary data, ethical approval was not required.

Bibliometric Analysis

Annual scientific production: The data were analyzed using Biblioshiny, a web-based application integrated with R (version 4.2.2; R Foundation for Statistical Computing, Vienna, Austria; https://www.R-project.org/). Biblioshiny facilitates the visualization and analysis of bibliometric data, focusing on sources, authors, conceptual structures (e.g., thematic maps), and documents (including author keywords). It provides a comprehensive set of indicators to evaluate the contributions of countries, authors, institutions, and journals. Publication output over the years was assessed using the "Annual Scientific Production" function which allows visualization of the number of publications per year. The analysis was configured to aggregate the data by publication year, ensuring that trends over time could be accurately represented. The output was displayed as a line chart, showing the total number of publications for each year in the dataset. This visualization allowed for the identification of trends, such as periods of growth, decline, or consistent output in AC research. The resulting chart and corresponding data were exported for documentation. The results were cross-checked with the dataset to confirm that all publication years were accurately represented and no records were omitted.

Local citations and source production: The "Local Citations" metric was selected to identify the top 10 most locally cited sources, representing the number of times sources were cited by articles within the dataset, while H-index values were sought from Scimago Journal & Country Rank website (www.scimagojr.com) to provide additional context on the impact and productivity of the sources. The "Source Production" metric was used to determine the top 10 sources based on the number of articles published. The results were visualized and exported as tables, and finally, the findings were cross-validated with the original dataset to ensure accuracy and consistency. This approach provided robust and interpretable results, showcasing the most impactful and prolific sources in the field.

Top 100 most-cited documents: The "Most-Cited Documents" function was selected to identify the articles with the highest citation counts. The analysis was configured to rank all articles in the dataset by total citation count. From the ranked list of cited documents, the top 100 articles with the highest citation counts were isolated. These articles were examined for additional details such as their publication years, journals, and authors to provide context for their impact. The list of the top 100 most-cited articles was exported from Biblioshiny. The data was cross-validated against the original dataset to ensure accuracy, confirming that all articles and citation counts matched the source data. The results were analyzed to provide insights into the most influential studies in AC research, highlighting significant contributions and their citation impact within the field.

Author Distribution Based on Sex

The distribution of male and female authorship among the top 100 most-cited articles was calculated, and it focused on the first, second, and last authorship positions, which are key contributors to the academic authorship hierarchy. The extracted authors were searched online using their affiliation details to confirm their sex. Sources such as institutional profiles, professional websites, and academic biographies were used to gather this information. This step ensured accurate sex classification, particularly for names that might not be clearly identifiable. This data was aggregated to count the total number of male and female authors in each of the three authorship positions across all 100 articles. The aggregated data were analyzed to determine the distribution of male and female authors in each position. The results were presented using a table to visualize sex distribution trends among key authorship roles. For second authorship, only articles with at least three authors were included in the analysis, as this position is not applicable to single- or two-authored papers. Similarly, for last authorship, only articles with at least three authors were considered, as the concept of a last author does not exist in single-authored papers and is equivalent to the second author in two-authored papers. In single-authored papers, the count was placed on the first author position. In two-authored papers, the second author position was treated as a senior role. For three-author papers, all three positions first, second, and last authorship were included in the analysis, ensuring a comprehensive evaluation of authorship roles.

Results

The distribution of the 718 publications retrieved from the database was as follows: 85.8% were articles, followed by reviews (11.8%), proceeding papers (3.8%), and early access documents (0.8%). The majority of these publications were written in English (97.6%), followed by German (0.8%), Spanish (0.4%) and Japanese (0.4%), French (0.3%), and Czech (0.1%), Polish (0.1%), and Turkish (0.1%).

Annual Scientific Production

**Figure 1:**
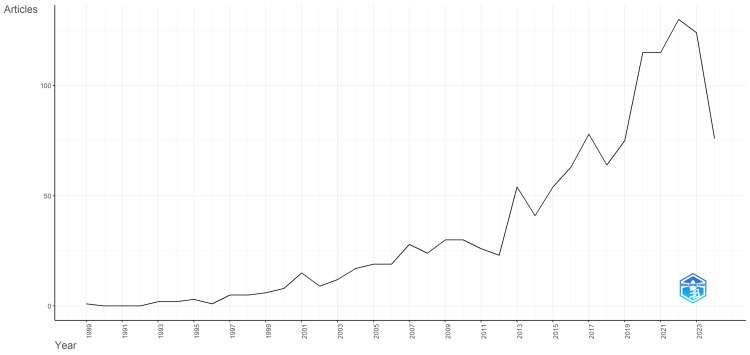
Number of scientific articles on awake craniotomy published per year from 1989 to 2024. The image was created by the authors using Biblioshiny.

Analysis showed low and relatively steady publication output from 1989 to around 2013, with only minor fluctuations in article counts (Figure 1). After 2013, there was a sharp increase in the number of articles published on AC, peaking in 2021 with over 60 publications. The rise may reflect advancements in surgical techniques, or growing interest in the clinical outcomes and benefits of AC, leading to a surge in related research. In the last couple of years (2022-2023), the number of articles appeared to have slightly declined but remained significantly higher than in previous decades. This pattern suggests growing research interest in AC, especially in recent years.

**Figure 2:**
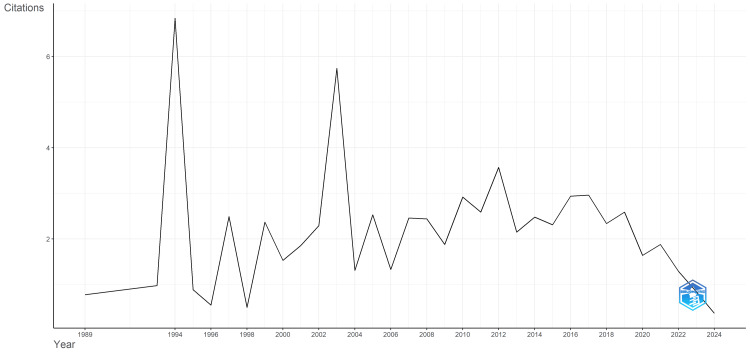
Average citations per year for published articles on awake craniotomy from 1989 to 2024. The image was created by the authors using Biblioshiny.

Analysis showed significant fluctuations in citation averages over the years (Figure 2). This trend may indicate that while early studies on AC gained considerable attention and citations, more recent publications have not been cited as frequently. The decline in recent years could be influenced by a variety of factors, including the saturation of research in the field, a shift in research focus, or changes in citation practices.

Local Citations and Source Production

An analysis of the most influential sources in AC was performed (Table 1). Regarding the top 10 locally cited sources, the *Journal of Neurosurgery* had the highest citations (2,611) and an H-index of 236, followed by *Neurosurgery* (2,000 citations, H-index 222) and *Acta Neurochirurgica* (820 citations, H-index 109). Notably, *Neuroimage* and *Brain* had high H-indices (418 and 380, respectively), reflecting their significant impact despite fewer citations. Publication activity ranked *World Neurosurgery* as the most prolific journal with 71 articles, followed by *Journal of Neurosurgery *(49 articles) and *Neurosurgery* (28 articles) (Table 2).

**Table 1:** Top 10 most local cited sources based on the total number of citations that each source has received on awake craniotomy studies.

Source	H-index	Citations
Journal of Neurosurgery	236	2611
Neurosurgery	222	2000
Acta Neurochirurgica	109	820
World Neurosurgery	115	736
Anesthesia & Analgesia	227	643
Journal of Neurosurgical Anesthesiology	71	531
Journal of Neuro-Oncology	163	440
Brain	380	432
NeuroImage	418	427
Anesthesiology	267	365

**Table 2:** Top 10 relevant sources based on the number of articles published on awake craniotomy

Source	Articles
World Neurosurgery	71
Journal of Neurosurgery	49
Neurosurgery	28
Journal of Neurosurgical Anesthesiology	26
Acta Neurochirurgica	24
Journal of Clinical Neuroscience	21
Clinical Neurology and Neurosurgery	18
British Journal of Neurosurgery	16
Journal of Neuro-Oncology	16
Cureus Journal of Medical Science	15

Top 100 Most-Cited Documents

For the top 100 most-cited articles in neurosurgery, the leading article, authored by Haglund et al. in 1994 [14] and published in *Neurosurgery*, had accumulated 377 citations, averaging 12.16 citations per year. This was followed by Taylor et al.'s article [15] in the *Journal of Neurosurgery* with 288 citations, and Hervey-Jumper et al.'s article [16] in the same journal, which received 261 citations with an impressive average of 26.10 citations per year (Table 3). The list featured a blend of foundational and recent works, predominantly published in high-impact journals such as the *Journal of Neurosurgery* and *Neurosurgical Focus*.

**Table 3:** The top 100 most-cited papers on awake craniotomy

Rank	Author	Source	TC	TC per Year	Normalized TC
1	Haglund et al. [14]	Neurosurgery	377	12.16	1.77
2	Taylor and Bernstein [15]	J Neurosurg	288	11.08	1.81
3	Hervey-Jumper et al. [16]	J Neurosurg	261	26.10	9.27
4	Szelényi et al. [17]	Neurosurg Focus	258	17.20	4.59
5	Bello et al. [18]	Neurosurgery	222	12.33	3.46
6	Lara-Velazquez et al. [19]	Brain Sci	195	24.38	7.37
7	Yetkin et al. [20]	Am J Neuroradiol	188	6.71	2.69
8	De Benedictis et al. [21]	Neurosurgery	182	12.13	3.24
9	Sacko et al [22]	Neurosurgery	179	12.79	4.24
10	Kim et al. [23]	Neurosurgery	175	10.94	6.19
11	Serletis and Bernstein [24]	J Neurosurg	174	9.67	2.72
12	Duffau et al. [25]	J Neurol Neurosur Ps	139	6.04	2.87
13	Nossek et al. [26]	J Neurosurg	134	11.17	3.81
14	Meyer et al. [27]	Mayo Clin Proc	133	5.54	2.40
15	Blanshard et al. [28]	Anesth Analg	127	5.29	2.29
16	Southwell et al. [29]	J Neurosurg	127	14.11	4.76
17	Sarang and Dinsmore [30]	Brit J Anaesth	123	5.59	2.03
18	Gupta et al. [31]	Clin Neurol Neurosur	121	6.72	1.89
19	Nossek et al. [32]	Neurosurgery	116	9.67	3.30
20	Bekker et al. [33]	Anesth Analg	103	4.29	1.86
21	Eseonu et al. [34]	Neurosurgery	101	12.63	3.82
22	Yamao et al. [35]	Hum Brain Mapp	101	9.18	3.01
23	Quiñones-Hinojosa et al. [36]	J Neurosurg	101	4.59	1.67
24	Ard et al. [37]	J Neurosurg Anesth	99	4.50	1.63
25	Whittle et al. [38]	Acta Neurochir	99	4.95	2.26
26	Boetto et al. [39]	World Neurosurg	94	9.40	3.34
27	Maldonado et al. [40]	J Neurosurg	94	6.71	2.22
28	Souter et al. [41]	J Neurosurg Anesth	92	5.11	1.44
29	Goettel et al. [42]	Brit J Anaesth	92	10.22	3.45
30	Manninen et al. [43]	Anesth Analg	91	4.79	2.70
31	Piccioni and Fanzio [44]	Minerva Anestesiol	90	5.29	3.42
32	Brown and Brown [45]	J Neurosurg Anesth	89	7.42	2.53
33	Bello et al. [46]	Neurosurgery	89	4.68	2.64
34	Khu et al. [47]	J Neurosurg	87	5.80	1.55
35	Mack et al. [48]	J Neurosurg Anesth	79	3.76	2.69
36	Stevanovic et al. [49]	Plos One	78	8.67	2.93
37	Hans et al. [50]	Anaesthesia	71	2.84	1.81
38	Gerritsen et al. [51]	Acta Neurochir-A	70	11.67	5.29
39	Hansen et al. [52]	Acta Neurochir	70	5.83	1.99
40	Bernstein [53]	Can J Neurol Sci	68	2.83	1.23
41	Herrick et al. [54]	Anesth Analg-A	68	2.43	0.97
42	Picht et al. [55]	Acta Neurochir	65	3.42	1.93
43	Costello et al. [56]	J Clin Neurosci	65	3.10	2.21
44	Chacko and Cormack [57]	Clin Neurol Neurosur	63	5.25	1.79
45	Rozet [58]	Curr Opin Anesthesio	63	3.71	2.40
46	Mäkelä et al. [59]	Hum Brain Mapp	62	2.58	1.12
47	Berkenstadt et al. [60]	J Neurosurg Anesth	60	2.50	1.08
48	Pereira et al. [61]	Acta Neurochir	59	3.69	2.09
49	Chang et al. [62]	J Neurosurg	59	7.38	2.23
50	Ard et al. [63]	Surg Neurol	58	2.90	1.32
51	Krieg et al. [64]	Bmc Neurosci	58	5.27	1.73
52	Duffau [65]	Neurosurg Rev	58	8.29	3.14
53	Manninen and Tan [66]	J Clin Anesth	57	2.48	1.18
54	Trinh et al. [67]	Neurosurgery	56	4.67	1.59
55	Lubrano et al. [68]	Neurosurgery	55	3.67	0.98
56	Flexman et al. [69]	J Neurosurg Anesth	55	11.00	4.97
57	Herrick et al. [70]	Anesth Analg	54	1.93	0.77
58	Saito et al. [71]	J Neurosurg	54	4.91	1.61
59	Gignac et al. [72]	Can J Anaesth	53	1.66	1.00
60	Frost and Booij [73]	Curr Opin Anesthesio	53	2.94	0.83
61	Fontaine et al. [74]	Brain	53	7.57	2.87
62	Papanicolaou et al. [75]	Epilepsia	52	4.73	1.55
63	Palese et al. [76]	Cancer Nurs	51	3.00	1.94
64	Wahab et al. [77]	Brit J Neurosurg	51	3.64	1.21
65	Gogos et al. [78]	J Neuro-Oncol	51	10.20	4.61
66	Lu et al. [79]	J Clin Neurosci	51	4.25	1.45
67	Chang et al. [80]	J Neurosurg	51	3.64	1.21
68	Sommer et al. [81]	Neurosurg Focus	51	4.25	1.45
69	Maldaun et al. [82]	J Neurosurg	50	4.55	1.49
70	Bilotta and Rosa [83]	Curr Opin Anesthesio	50	3.13	1.77
71	Brennan et al. [84]	Neuroimage	50	2.78	0.78
72	Roland et al. [85]	Epilepsy Behav	49	3.27	0.87
73	Saito et al. [86]	Neurol Med-Chir	49	4.90	1.74
74	Kemp et al. [87]	World Neurosurg	49	3.77	2.35
75	Venkatraghavan et al. [88]	Can J Anesth	49	5.44	1.84
76	Kim et al. [89]	Neurosurgery	49	3.27	0.87
77	Breshears et al. [90]	J Neurosurg	49	4.90	1.74
78	Grossman et al. [91]	Ann Surg Oncol	48	4.00	1.36
79	Klimek et al. [92]	Anaesthesia	48	2.29	1.63
80	Haglund et al. [93]	J Neurosci	48	1.55	0.23
81	Balogun et al. [94]	J Clin Neurosci	47	4.27	1.40
82	Cohen-Gadol et al. [95]	J Neurosurg	47	2.14	0.78
83	Garavaglia et al. [6]	J Neurosurg Anesth	46	4.18	1.37
84	Gonen et al. [96]	J Neurosurg	46	4.18	1.37
85	Alimohamadi et al. [97]	World Neurosurg	46	5.11	1.73
86	Goebel et al. [98]	Neurosurgery	46	3.07	0.82
87	Motomura et al. [99]	J Neurosurg	46	4.18	1.37
88	Milian et al. [100]	Acta Neurochir	45	4.09	1.34
89	Costello et al. [101]	Brit J Anaesth	45	2.25	1.03
90	Delion et al. [102]	World Neurosurg	45	4.50	1.60
91	Carrabba et al. [103]	Minim Invas Neurosur	45	2.65	1.71
92	Santini et al. [5]	J Neurosurg Anesth	45	3.46	2.16
93	Klijn et al. [104]	J Neurosurg	45	3.75	1.28
94	Olsen [105]	Eur J Anaesth	45	2.65	1.71
95	Leuthardt et al. [106]	Neurosurgery	44	3.14	1.04
96	Cannestra et al. [107]	Neurosurgery	44	2.10	1.50
97	Costello et al. [108]	J Neurosurg Anesth	43	2.05	1.46
98	Dziedzic and Bernstein [109]	Expert Rev Neurother	43	3.91	1.28
99	Meng et al. [110]	J Neurosurg Anesth	43	4.30	1.53
100	Eseonu et al. [111]	World Neurosurg	43	5.38	1.62

Author Distribution Based on Sex

The distribution of author positions based on sex in a bibliometric analysis of the top 100 most-cited articles was calculated and presented in Table 4. It focused on the first, second, and last author roles. In all the author positions considered, most individuals were male. These results highlight sex disparities in authorship, especially in the senior authorship position.

**Table 4:** Distribution of male and female authorship positions among the top 100 most cited articles.

	Male		Female		
Position	Absolute	Relative	Absolute	Relative	Total
First author	77	77%	23	23%	100
Second author	60	68.97%	27	31.03%	87
Last author	84	87.5%	12	12.5%	96

Discussion

Annual Scientific Production and Local Citation

The trend of scientific production on AC over the past few decades reflects shifts in research interest. From 1989 to around 2013, the number of publications remained relatively low with minor fluctuations. This period likely represents the foundational stage of AC research, during which the feasibility and safety of the procedure were probably being explored in a limited number of centers. One of the first large studies that described the anesthetic experience with AC was published by Archer et al. in 1988 [112]. The low and steady scientific output on AC from 1989 to around 2013 can be partially attributed to its limited geographic adoption and the relatively slow dissemination of the practice globally. AC was primarily practiced in North America from the 1980s and only gradually expanded to Europe and later to Asia [113]. Its introduction into Asia, particularly after the early 2000s, holds significance due to the region's large patient population and the relatively low resource requirements of AC, making it a feasible and cost-effective surgical option. In the late 1990s, while case reports began emerging from Asian countries like Japan, India, and Thailand, these were largely published in local neurosurgical journals, limiting their international visibility and impact on global scientific output [114]. Additionally, countries such as China and Indonesia only introduced AC between 2003 and 2007, further reflecting the delayed adoption of the technique in certain regions [114]. 

The period between 1989 and 2013 can also be linked to the gradual evolution and dissemination of this technique. AC, though rooted in ancient practices such as trepanation for seizures and other ailments, only emerged in its modern form with the application of brain mapping for the surgical removal of epileptic foci. Over time, advancements expanded its use to the resection of tumors in functional cortical areas and, more recently, to supratentorial tumors without selective involvement of the eloquent cortex [114]. The sharp increase in publications after 2013 aligns with the broader global adoption of AC, advancements in surgical and anesthetic techniques, and a growing recognition of its benefits in preserving neurological function. As the technique gained traction across continents, particularly in Asia, it likely spurred more widespread research collaborations and higher visibility in international journals, contributing to the surge in publication output.

Top 100 Most-Cited Articles 

The analysis of the top 100-most cited articles highlights the field's evolution and the impact of pioneering studies in enhancing surgical, anesthetic, and neuro-oncological techniques. The highly cited Haglund et al.'s study, "Cortical Localization of Temporal Lobe Language Sites in Patients with Gliomas," is foundational in AC research due to its focus on accurately mapping language areas to preserve function during glioma surgery [14]. By using AC for direct cortical stimulation in conscious patients, this study offered a crucial approach to minimize language deficits post surgery, which has since shaped protocols for balancing tumor resection with functional preservation. Its insights into language site localization have not only improved patient outcomes but also established a standard for subsequent innovations in brain mapping and neurosurgical techniques. Haglund et al.'s was the oldest article on the list [14], followed by Taylor and Bernstein [15], giving these studies considerably more time to accumulate citations compared to more recent publications. By analyzing 200 cases, Taylor and Bernstein [15] demonstrated that AC enabled maximal tumor removal, minimized neurological deficits, and reduced ICU and hospital stays. They established AC with brain mapping as a standard approach for safely resecting supratentorial intra-axial tumors.

More recent publications, like that of Hervey-Jumper et al. [16] and Lara-Velazquez et al. [19], showed a significant increase in the average citations per year. Hervey-Jumper's work emphasized advancements in intraoperative mapping and imaging. Hervey-Jumper et al.'s article [16] was highly cited perhaps due to its comprehensive analysis of AC techniques and outcomes for glioma resection over a 27-year period. They recorded improvements in seizure management that have also contributed to reducing complications. Szelényi et al. contributed to a growing body of literature on monitoring techniques that help to mitigate neurological risks [17]. The article provided a comprehensive review of intraoperative electrical stimulation techniques for AC, especially in the context of low-grade glioma surgery. By including participant's personal experiences and discussions with the European Low-Grade Glioma Network, the article offered practical guidelines on brain mapping that have helped standardize approaches across neurosurgical centers. Similarly, Bello et al. addressed the intraoperative use of neurophysiological monitoring, which has become a cornerstone in the field, helping surgeons preserve patient motor and cognitive functions during tumor resection [18]. The article by Lara-Velazquez et al. provided an extensive review of advanced surgical approaches for glioblastoma (GBM), the most aggressive primary brain tumor [19]. This work outlined innovative techniques, such as AC, fluorescence-guided surgery, laser interstitial thermal therapy, and intraoperative mass spectrometry, which could have contributed to it being the sixth highly cited article. 

Among anesthetic-focused studies, Yetkin et al. [20] and Kim et al. [23] provided foundational insights into the safe administration of anesthesia during AC procedures. Yetkin et al’s work, helped shape protocols for neuroanesthesia, while Kim et al.’s article focused on optimizing patient comfort and managing airway and consciousness levels during surgery. Souter et al. also provided insights into anesthetic management, with an emphasis on minimizing systemic effects, which is critical for the high-risk patient populations often undergoing ACs [41].

De Benedictis et al.'s study was highly cited probably for demonstrating that AC with intraoperative mapping enables a more extensive and safer resection of low-grade gliomas in eloquent areas compared to traditional surgery [21]. By directly comparing outcomes from surgeries with and without awake mapping in the same patients, the study highlighted the effectiveness of intraoperative brain stimulation in maximizing tumor removal while preserving critical brain functions. The technique allowed surgeons to perform resections based on functional boundaries, reducing postoperative morbidity and enhancing quality of life. Although the study involved a limited number of cases, the growing adoption of awake brain mapping reflects its increasing safety, feasibility, and reproducibility. The authors recommended that there is a need to expand this approach through multidisciplinary and multicenter studies with standardized data collection to facilitate more accurate comparisons across treatment methods. Sacko et al. compared AC with intraoperative brain mapping to surgery under general anesthesia for the resection of supratentorial lesions [22]. By analyzing outcomes in a large cohort, the study demonstrated that AC significantly improved neurological outcomes and the extent of resection for tumors near functional brain regions. AC patients also had shorter ICU and hospital stays, fewer complications, and better functional preservation than those treated with GA. This evidence-based support for AC’s advantages, backed by its large sample size and clear comparative findings, has made it an influential reference in neurosurgery.

Comparative studies between awake and traditional craniotomy highlight significant advantages of AC in specific aspects of patient care. Sacko et al. [22] and Vigren et al. [115] reported that AC is both safe and effective, with Sacko observing improved neurological outcomes and resection quality, while Vigren notes its feasibility in patients previously deemed inoperable. Hol et al. support these findings by demonstrating that AC produces fewer alterations in plasma amino acid profiles, suggesting a potentially lower metabolic impact compared to traditional craniotomy [116]. Additionally, Eseonu et al. compared two sedation methods for AC and found that both are safe and effective, with monitored anesthesia care yielding shorter operative times [34]. Zelitzki et al. reported better neurological outcomes, early postoperative motor function, and shorter hospital stays with AC [117]. Notably, these studies did not extensively assess long-term functional status and quality of life, or the importance of patient-specific factors in optimizing these outcomes, suggesting the need for further research focused on long-term quality of life and functional recovery in patients undergoing awake versus traditional craniotomy. While AC shows promising results, some studies present mixed findings, for example, Gravesteijn et al. reported comparable outcomes in resection extent, neurological status, and survival rates between the two methods [118]. 

Several other top-ranking studies contributed valuable insights into the use of AC across diverse neurological conditions. For instance, two different studies by Nossek et al. examined AC applicability for brain tumors and epilepsy surgery [26,32]. These articles discussed the growing consensus on AC benefits in complex neurological cases and highlighted the importance of developing specialized protocols for patient monitoring and comfort. Duffau’s et al.'s study is highly influential in advocating for functional preservation during glioma surgery, helping surgeons to achieve maximal tumor resection with minimal impact on neurological function [25]. Their study laid the groundwork for the now-common practice of mapping critical functional areas intraoperatively.

Other noteworthy studies in the top 20 included those by Meyer et al. [27] and Blanshard et al. [28], which collectively emphasized safety and anesthetic considerations that minimize patient discomfort and physiological disturbances during awake procedures. Both studies discussed protocols that ensure patient safety while allowing real-time neurological assessments. More recent studies like those by Southwell et al. [29] and Eseonu et al. [34] demonstrated the evolution of AC protocols to accommodate newer technologies like neuronavigation and advanced neuroimaging. Southwell’s work is particularly notable for investigating AC outcomes in a more diverse patient population, reflecting the procedure’s expanding application beyond traditional glioma cases.

Yamao et al. [35] and Quinones-Hinojosa et al. [36] contributed to the understanding of brain mapping techniques, which are integral to AC success. These articles also underscore the procedure's critical role in neurological preservation. Together, these top 20 publications collectively reinforce the procedure's value in neurosurgical practice, highlighting ongoing improvements in technique, patient safety, and functional outcomes. The continued integration of new technologies and collaborative efforts across surgical and anesthetic fields will likely further elevate the role of AC in neuro-oncology and beyond, solidifying its status as a crucial tool for modern neurosurgery.

Author Distribution Based on Sex

The analysis of authorship demographics categorized by sex revealed that the majority of the first authors in the top 100 most-cited articles were male, compared to only 23% being female. The sex distribution is even more skewed in the last authorship position, with just under 14% of authors being female. These findings align with the overall proportion of females in the field of neurosurgery, who constitute 19% of all board-certified neurosurgeons globally [119]. The most pronounced disparity in the number of female neurosurgeons was observed in Europe, where the highest proportion was in Italy and the lowest in Cyprus and Kosovo, at 36% and 0%, respectively [120].

The study by Aslan et al., which analyzed 3,247 original articles over a 15-year period, assessed sex distribution among authors [121]. The proportion of female authors in the first author position showed a notable increase from 12% to 16%, but senior authorship positions saw a decline from 11.7% in 2003 to 10.5% in 2018 [121]. Although there is a general upward trend in female neurosurgeons participating in research, this progress remains insufficient, as the sex disparity is still significant. Furthermore, there is a striking sex gap in academic neurosurgery, with 92.55% of leadership positions occupied by male authors [122]. These findings highlight the remarkable underrepresentation of females in both non-academic and academic neurosurgery.

Several factors have been identified as contributing to this significant sex disparity, including cultural and social differences, workplace harassment, and challenges in maintaining a work-life balance [123]. According to Zeitlberger et al., female neurosurgeons were more likely to experience discrimination from colleagues as well as from patients and their families [124]. Among respondents, 88% of females and 38.1% of males reported experiencing sex inequity at work. Female neurosurgeons were found to be 4.3 times more likely to face discrimination from colleagues and 3.6 times more likely to face discrimination from patients or relatives. Additionally, 78% of females stated that they felt they had to work harder than their male counterparts to achieve the same level of recognition. Lulla et al. conducted a comprehensive review of factors contributing to sex disparities [120]. Lifestyle was identified as a major barrier preventing females from entering neurosurgical training. Female respondents were also less likely to be married or have children compared to their male colleagues. In Japan, a survey of women leaving neurosurgical positions indicated that the majority cited difficulty balancing a neurosurgical career with motherhood as the primary reason for leaving [125]. Career satisfaction was another key factor in the sex gap. Female neurosurgeons reported lower career fulfillment compared to their male counterparts and were less likely to choose this career again [125].

Overall, more women are entering neurosurgical training each year. While progress has been slow, the trend toward closing the sex gap and achieving equity in opportunities and career goals is steadily improving. However, significant challenges remain, with discrimination, career satisfaction, and work-life balance being the most critical issues. To address these disparities, cultural change is necessary to ensure that the achievements of female neurosurgeons are recognized by their male counterparts, fostering a supportive and inclusive work environment.

Technological Advancements in AC

Innovative tools for brain mapping: A recent study focused on developing a digital platform, map-OR, to facilitate intraoperative language testing and collaborative data sharing for ACs. The mixed-methods research included international surveys with AC teams from 14 countries, synthesis of guiding principles, and risk assessment. Six technologies were identified for language mapping, utilizing portable devices and virtual reality headsets. The study also highlighted factors influencing the adoption of surgical technologies. Survey data showed that over half of the teams used digital language testing methods, primarily tablet computers and Microsoft PowerPoint. The study identified four key risks, with software and connectivity issues as primary concerns, establishing a structured framework for digital language testing and data sharing in AC [126].

Enhanced intraoperative language monitoring: A systematic review of 102 studies (up to July 2020) examined speech and language errors in glioma patients undergoing AC with direct electrical stimulation (DES). The review found that although cortical areas were more frequently studied, approximately 40% of errors were linked to subcortical regions. Patterns of speech and language error localization aligned with the dual-stream language processing model and the Dutch Linguistic Intraoperative Protocol (DuLIP). Additional locations for specific language functions, including motor speech, phonology, reading, and writing, were also identified, leading to an updated DuLIP model. This model can guide task selection during AC, enhancing intraoperative monitoring and postoperative language outcomes [127].

Recent advancements in imaging and procedural techniques are enhancing the precision and outcomes of AC. Virtual reality (VR) and augmented reality (AR) are increasingly utilized to aid intraoperative navigation and cognitive assessment. VR enables surgeons to visualize detailed, three-dimensional (3D) anatomical structures in real time and evaluate neurological functions during AC, offering insights into cognitive domains such as attention, memory, and language processing [128]. AR, meanwhile, overlays critical imaging data, like tumor boundaries and adjacent structures, onto the surgical field, reducing intraoperative cognitive load and improving the precision of resection in eloquent areas [128].

Diffusion tractography (DT) is another innovation supporting AC. A study of 100 procedures integrating DT with intraoperative stimulation found that DT predictions aligned closely with intraoperative findings, demonstrating high sensitivity (92.2%) in mapping spatial relationships but moderate specificity in predicting functional outcomes. DT was particularly valuable in assessing postoperative recovery potential, helping to identify tract preservation and forecast recovery trajectories in patients with transient deficits [129].

The combination of AC with intraoperative MRI (iMRI) has also shown significant benefits in maximizing tumor resection. In a review of 33 cases, iMRI identified residual tumors in 64% of patients, enabling additional resection and resulting in a maximal extent of resection (>90%) in half of these cases [129]. This approach proved especially beneficial for complex tumors in challenging locations, such as the insular lobe, where it facilitated an increased resection extent, potentially improving patient outcomes. For arteriovenous malformations (AVMs) near language areas, AC combined with cortical and subcortical mapping enables safe resection while preserving language function. In a series of AVM resections, cortical and subcortical stimulation minimized language deficits, with no permanent neurological complications reported, underscoring the efficacy of this approach in complex cases [130].

Challenges and Limitations

AC presents unique physical and psychological challenges for both patients and the surgical team. Patients may experience anxiety, discomfort, and postoperative emotional effects. While studies such as that by Tang and Tan [131] emphasize the importance of close communication and management strategies to address potential complications like intraoperative nausea or loss of cooperation, others, such as that by Hejrati et al. [10] and Wajer et al. [9] report that although some patients experience anxiety or depressive symptoms postoperatively, these do not significantly exceed preoperative levels. Psychological support tailored to each patient is crucial, as Starowicz-Filip et al. highlight the occasional anxiety patients may feel about the procedure [132]. 

The surgical team must navigate technical challenges, including maintaining patient comfort and cooperation during awake procedures while managing risks like airway loss or seizures [131]. Introducing specialized personnel, such as intraoperative monitoring technicians, adds complexity, but with clear roles, they enhance patient outcomes [133]. Although resource-intensive, AC has been successfully adapted for low- and middle-income countries, improving access to neurosurgery [134]. Nevertheless, limitations due to specialized equipment and personnel needs, such as neuropsychologists and neurophysiologists, may restrict its widespread implementation in resource-constrained settings [135]. Nonetheless, evidence supports its feasibility and effectiveness even with limited resources, yielding favorable functional and oncologic outcomes and benefits in specific cases, like cerebral arteriovenous malformation resection [136,137]. Similarly, Bharadwaj et al. reported that while some patients experienced discomfort, anxiety, or fear, most coped well when adequately informed and supported, highlighting the need for patient-centered strategies to enhance comfort and manage anxiety [138]. In South America, AC adoption has shown benefits like reduced hospital time, faster recovery, and lower morbidity, yet infrastructure limitations, lack of equipment, and workforce shortages continue to challenge its expansion [138]. Addressing these barriers is essential to maximizing AC's impact on neurosurgical care in the region.

Limitations of the Study

This study is limited by the fact that the data were retrieved from a single database (Web of Science Core Collection), which may not capture all relevant publications or fully represent the field, potentially excluding important research. Moreover, the study relies on citation counts and the number of publications as a measure of impact, which may not fully reflect the quality or influence of a study, as newer articles may not yet have accumulated significant citations. Although the study focused on publications related to AC, the results may not account for all variations in terminology or research scope within the broader field. Future studies should expand data sources and consider alternative metrics for assessing research impact

## Conclusions

This bibliometric analysis of the top 100 most-cited articles on AC brought out the significant growth and evolution of AC neurosurgical technique, particularly after 2013. AC has been established as the gold standard for tumor resection near eloquent brain regions, enabling maximal resection while preserving critical functions like speech and motor control. Technological advancements, including intraoperative MRI, diffusion tractography, and augmented/virtual reality, have refined AC by improving surgical precision and patient outcomes. Despite these advancements, challenges persist in patient selection, intraoperative management, and addressing psychological impacts. The study highlighted significant gender disparities in authorship, reflecting broader inequities in neurosurgery and academic medicine. Future research should focus on long-term outcomes, advanced mapping techniques for complex cognitive functions, and adapting AC for resource-limited settings. Overall, AC continues to revolutionize neurosurgical care, balancing oncological efficacy with functional preservation.
